# Diagnostic role of interleukin -33 in the differentiation of pleural effusions especially tuberculous and malignant effusions

**DOI:** 10.1186/s12890-019-0874-y

**Published:** 2019-06-25

**Authors:** Abdel-sadek Hamed Al-aarag, Mohammad Hussein Kamel, Eman Ramadan Abdelgawad, Shaimaa Magdy Abo-Youssef, Hany Hussein Moussa, Marwa Elsayed Elnaggar, Rasha Mohammad Hendy, Koot Ahmad Diab

**Affiliations:** 1 Faculty of medicine, Benha University, Banha city, Qalubia Province 13518 Egypt; 2 Faculty of medicine, Kafrelsheikh University, fifth foutoh salam street, Banha city, Qalubia Province 13518 Egypt

**Keywords:** Pleural effusion, Tuberculosis pleurisy, Malignant pleural effusion

## Abstract

**Background:**

Tuberculous pleurisy and malignancy are two of the most common causes of pleural effusion. IL-33 is expressed in the epithelial lining and endothelial cells and is released after cell damage; it is proposed to have an essential role in sensing damage in various infectious and inflammatory diseases. This work aimed to determine the diagnostic role of IL-33 in pleural effusions.

**Methods:**

One hundred seventeen patients with pleural effusions of different etiologies had a quantitative measurement of IL-33 in their pleural effusion and serum samples by ELISA technique.

**Results:**

The concentrations of IL-33 (mean ± SD) in tuberculous pleural effusion (TPE) group (22.5 ± 0.90 ng/l) were significantly higher than that of malignant pleural effusion (MPE) group (14.6 ± 2.35 ng/l; *P* <  0.001). There is no significant difference between the serum levels of IL-33 in (TPE) group and (MPE) group (*P* >  0.05). The concentrations of IL-33 in the pleural effusions were significantly correlated to that of the serum concentrations in each group (TPE: *r* = 0.848, *P* = < 0.001; MPE: *r* = 0.881, < 0.001) and pleural ADA in patients with tuberculous pleural effusions, (*r* = 0.38, *P* <  0.001). The cut-off value of pleural IL33 for (TPE) was 19.16 ng/l, with a sensitivity of 91.7%, a specificity of 96.4%. The cutoff point of a pleural/ serum IL-33 ratio for the diagnosis of TPE was > 1.4 with a sensitivity of 91.7% and specificity of 100% while for the determination of (MPE) was < 0.9 with a sensitivity of 83.3% and specificity of 96.4%.

**Conclusion:**

IL-33 level may serve as a novel biomarker to differentiate pleural effusions, especially tuberculous from malignant effusions.

## Summary at a glance

One hundred seventeen patients with pleural effusions of different etiologies had a quantitative measurement of IL-33 by ELISA technique in pleural effusion and serum samples of the patients. Pleural IL-33 is a novel biomarker for diagnosis and differentiation of different forms of pleural effusion especially tuberculous from malignant effusions.

## Background

Differentiating pleural effusions especially tuberculous pleural effusion from malignant pleural effusion is still a clinical challenge since they have similar clinical or laboratory manifestations and sometimes lack of pathological or etiological evidence [1]. Given the difficulties in ascertaining the etiology of pleural effusions from the simple fluid analysis, the need to new biomarkers emerges [2]. Cytokines as Interferon (IFN)-γ and interleukin (IL)-1, − 6, − 16 and − 17 released from lymphocyte activation have proven to have differential and prognostic significance in tuberculous and malignant pleural effusions [3]. Interleukin 33 (IL-33) is a cytokine of the IL-1 superfamily; it previously named as the nuclear factor of high endothelial venules. It acts intracellularly as a nuclear factor and extracellularly as a cytokine. It induces helper T cells, mast cells, eosinophils, and basophils to produce type 2 (Th2) helper cell cytokines [4, 5]. However, IL-33 is also capable of modulating type 1 helper (Th1) cell cytokine responses through inducing potent CD8 positive T cell responses in response to replicating, prototypic RNA and DNA viruses in mice [6] Some investigations showed that IL-33 expression increased in the pleural space of patients with tuberculous pleural effusion which induced by IFN-γ and tumor necrosis factor (TNF) [7]. Tumor development results in down-regulation of IL-33 in epithelial cells but up-regulation of IL-33 in the tumor stroma and serum. IL-33 expression in tumor cells increases immunogenicity and promotes type 1 antitumor immune responses [8].

### Patients and methods

A prospective study carried on 117 patients with pleural effusions of different etiologies; 72 (61.5%) male and 45 females (38.5%) and their ages ranged from 19 to 79 years (mean; 51.9 ± 14.6), admitted at Chest Department, Benha University Hospital during the period from September 2016 till August 2017. Ethical research approval from the institutional ethical committee and informed written consent from patients obtained. Patients classified according to their final diagnosis into four groups:

- Group I: Included 36 cases (30.8%), (18 males & 18 females) of tuberculous pleural effusion; their ages ranged from 30 years to 65 years with a mean age of 44.3 ± 9.81 years.
- Group II: Included 33 cases (28.2%), (18 males & 15 females) of malignant pleural effusion secondary to mesothelioma (18 cases), metastatic adenocarcinoma (15 cases); their age ranged from 19 years to 70 years (mean 54.9 ± 14.6 years).
- Group III: Included 24 cases (20.5%), (15 males & 9 females) of parapneumonic pleural effusion; their age ranged from 26 years to 75 years with a mean age of 48.8 ± 17.13 years.
- Group IV: Included 24 cases (20.5%), (21 males & 3 females) of transudative pleural effusion secondary to heart failure (3 cases), renal failure (3 cases) and liver cell failure (10 cases); their age ranged from 45 years 79 years with a mean age of 61.7 ± 13.61 years.

### Inclusion criteria

Exudative pleural effusion was diagnosed first by using modified Light’s criteria suggesting that pleural effusion is an exudate if effusion protein/serum protein ratio greater than 0.5 and or effusion lactate dehydrogenase (LDH)/serum LDH ratio greater than 0.6 [9], Table 1.

**Table 1:** demographic data and routine biochemical analysis of pleural effusions

GROUP	Age (years)	Sex		PH	Protein gm/dl	P/S^*^ protein ratio	LDH	P/S^*^ LDH ratio	ADA^*^(U/L)
		Male	female						
TB No = 36	44.3 ± 9.81	18	18	7.32 ± 0.08	4.4 ± 0.8	> 0.5	680 (330–2322)	> 0.6	81.6 ± 29.4
Malignancy No = 33	54.9 ± 14.6	18	15	7.33 ± 0.06	4.1 ± 1.2	> 0.5	560 (151–2218)	> 0.6	–
Para-pneumonic No = 24	48.8 ± 17.13	15	9	7.30 ± 0.09	4.0 ± 1.3	> 0.5	502 (133–8238	> 0.6	–
Transudative No = 24	61.7 ± 13.61	21	3	7.40 ± 0.05	2 + 0.8	< 0.5	142 (100–199)	< 0.6	–

^*^P/S pleural / serum ratio, ^*^ADA Adenosine Deaminase

### Group I (tuberculous pleural effusions): (I &II Plus any of IV or V)

I. Exudative pleural effusion identified.
II. The pleural effusion had an adenosine deaminase (ADA) concentration > 40 U/l [10]
III. Positive tuberculin skin test [11].
IV. Identification of acid-fast bacilli in the pleural effusion examination
V. Thoracoscopic or Abram’s needle pleural biopsy revealed caseating granuloma [12].

### Group II (malignant pleural effusion)

Symptomatic and rapidly accumulating pleural fluid (moderate to massive effusions occupying more than half of a hemithorax), and it had a chemical analysis proved to be exudative. Cytological examination revealed exfoliative malignant cells or confirmed by immunohistochemistry or histopathological examination of the biopsies obtained by different methods of biopsy [13].

### Group III (parapneumonic effusion)

Parapneumonic effusion is any pleural effusion secondary to pneumonia or lung abscess. The diagnosis considered when there was a clinical manifestation of pneumonia, radiological evidence of consolidation associated with effusion and analysis of this fluid. Uncomplicated parapneumonic effusion is not purulent, and yields negative results on Gram stain and culture, it has pH higher than 7.2, glucose more significant than 40 mg/dl and lactate dehydrogenase (LDH) lower than 1000 IU/l. While complicated parapneumonic effusion is purulent, and yields positive on either Gram stain or culture, with pH lower than 7.0, glucose lower than 40 mg/dl and LDH greater than 1000 IU/l [14].

### Group IV (transudative pleural effusion)

A- Patients with congestive heart failure (3 cases) diagnosed by the presence of the following evidence [15].

i. Chemical examination of the pleural fluid aspirate revealed transudative nature.
ii. Clinical symptoms and signs of heart failure; exertional dyspnea, orthopnea, paroxysmal nocturnal dyspnea, lower limb edema together with tachycardia or ventricular gallop and bilateral basal inspiratory crepitations.
iii. Cardiomegaly proved clinically, radiologically, or by echocardiography.
iv. Echocardiographical evidence of cardiac dysfunction (low left ventricular ejection fraction percentage)

B- Patients with liver cirrhosis and liver cell failure: (10 cases) were diagnosed according to the following criteria [16]:

i. Chemical examination of the pleural fluid aspirate revealed transudative nature.
ii. Clinical and laboratory evidence of hepatic injury, hypoalbuminemia,
iii. Portal hypertension guided by ultrasonographic examination.

C- Patients with renal failure (3 cases) diagnosed according to the following criteria [16].

i. Chemical examination of the pleural fluid aspirate revealed transudative nature.
ii. Clinical and laboratory evidence of renal impairment.
iii. Ultrasonographic evidence of renal affection.

Exclusion criteria: [17].

Patients with any of the following criteria excluded from the present study:

i. Treatment with antituberculous therapy or anticancer therapy
ii. The use of glucocorticoid and other anti-inflammatory medication.

All patients subjected to the following:

i. Thorough medical history and physical examination,
ii. Routine laboratory investigations: CBC, ESR, coagulation profile, fasting, and two hours post-prandial blood glucose. Liver and kidney functions tests. Serum total proteins & LDH.
iii. Radiological examination: Plain chest X-ray (PA and lateral views), CT scans of the chest, abdominal U/S, and echocardiography whenever needed.
iv. Tuberculin skin test in T.B suspected cases: Using the Mantoux method.
v. Sputum examination for acid-fast alcohol fast bacilli (AFB) by ziehl –neelsen stain in T.B suspected cases.
vi. Diagnostic thoracocentesis with pleural sample centrifugation at 5000 rpm for 10 min and stored at (− 80 °C) until used for the assay.
vii. Collection and processing of the pleural fluid samples; Physical, chemical, bacteriological, cytological examination, and quantitative measurement of pleural fluid IL-33 using ELISA technique.
viii. Pleural biopsies: were taken for patients in groups (I and II) by either Abram’s needle (3 cases, 2.5%) or thoracoscope (63 cases, 54%)
ix. Venous blood samples: For quantitative estimation of serum IL-33 by ELISA technique.
x. Collection and processing of the blood samples: Blood samples (3 cc) taken from the patients. Allowed to clot for 30 min and centrifugation separated the sera at about 5000 rpm for 10 min, sera were collected in pyrogen-free tubes and stored at (− 80 °C) until used for the assay.
xi. IL-33 assay: Quantikine Human IL-33 Immunoassay is a 4.5-h solid phase ELISA (Quantikine® ELISA, R&D Systems China Co., Ltd) used to assay IL-33 in pleural and serum samples of our patients. It uses microplate pre-coated with a polyclonal antibody specific for human IL-33. Standards and patient samples were pipetted into the wells, so if any IL-33 present in the samples would be bounded to the particular polyclonal antibodies. The wells were washed to remove away any unbound substances. A substrate solution was then added to the wells to check color development in proportion to the amount of bounded IL-33, and then the intensity of the color is measured.

### Statistical analysis

SPSS version 16 software (Spss Inc., Chicago, ILL Company) used to analyze the collected data, where the unequivocal data were given as number and percentages while quantitative data expressed as mean ± standard deviation, and range, using Shapiro-Wilks test for normality, assuming normality at *P* >  0.05, also paired “t” test, ANOVA and Person’s correlation (r) as proved to be normally distributed. ROC curve was used to detect cut off values of IL-33 in the prediction of tuberculous pleural effusion. The level of significance in this work started below 0.05 (*P* <  0.05 was considered significant) [18].

## Results

The pleural fluid IL-33 in all the studied groups level (14.14 ± 6.491 ng/l) was significantly higher of that of the corresponding serum IL-33 levels (12.6 ± 5.29 ng/l; *P* <  0.01). The mean pleural IL-33 levels of tuberculous group (22.5 ± 0.9), parapneumonic group (7.33 ± 0.72 ng/l) and transudate group (7.53 ± 0.38 ng/l) were significantly higher than the corresponding serum levels (13.9 ± 0.85 ng/l; *P* < 0.001), (6.22 ± .76 ng/l; *P* < 0.001) and (6.10 ± .42 ng/l; *P* < 0.001), respectively. However, serum IL-33 levels in the malignant group (13.5 ± 1.98 ng/l) were higher than the corresponding pleural level (11.9 ± 2.35 ng/l; *P* < 0.01), Table 2.

**Table 2:** Comparison between pleural and serum IL-33 levels in all studied groups, tuberculous, malignant, parapneumonic and transudative groups

Types of pleural effusion		Range	Mean (ng/l)	Paired “t.”	P
Pleural IL-33 of all studied group		6.44–23.95	14.14 ± 6.491	2.7	0.01 (S)^*^
Serum IL-33 of all studied group		5.23–20.32	12.06 ± 5.290		
Tuberculous *N* = 36	Pleural IL-33	21.00–23.95	22.5 ± 0.90	22.3	< 0.001 (HS)**
	Serum IL-33	12.46–15.11	13.9 ± 0.85		
Malignant *N* = 33	Serum IL-33	15.21–20.32	13.5 ± 1.98	10.5	< 0.01 (S)*
	Pleural IL-33	10.32–17.32	11.9 ± 2.35		
Parapneumonic *N* = 24	Pleural IL-33	6.44–8.43	7.33 ± 0.72	15.5	< 0.001 (HS)**
	Serum IL-33	5.23–7.21	6.22 ± 0.76		
Transudative *N* = 24	Pleural IL-33	7.12–8.21	7.53 ± 0.38	10.5	< 0.001 (HS)**
	Serum IL-33	5.38–6.78	6.10 ± 0.42		

*TST* Tuberculin Skin Test ^*^S significant, ^**^HS highly significant

Shows that the pleural IL-33 level is significantly higher than the serum IL-33 level in all studied groups. Pleural IL-33 is significantly higher than the serum level in tuberculous, parapneumonic, and transudative groups; while serum IL-33 is significantly higher than the pleural level in the malignant group

The concentrations of IL-33 in the pleural effusion positively correlated with that of the corresponding serum samples in all patients of the study groups (*r* = 0.677, *P* < 0.001). The concentrations of pleural IL33 in tuberculous, malignant and parapneumonic pleural effusion groups were also positively correlated with their corresponding serum IL33 level (*r* = 0.848, *P* < 0.001; *r* = 0.881, *P* < 0.001; and *r* = 0.965, *P* < 0.001) respectively. Also, Pleural IL-33 positively correlates with pleural ADA in patients with tuberculous pleural effusions, (*r* = 0.38, *P* < 0.001), Table 3.

**Table 3:** Correlation between serum and pleural IL-33 levels in different studied samples

	Serum IL-33 (ng/l)									
	All groups		TPE		MPE		Parapneumonic		Transudate	
	R	p	r	P	R	P	r	P	R	P
Pleural IL-33 (ng/l)	0.677	< 0.001 (HS)	0.848	< 0.001 (HS)	0.881	< 0.001 (HS)	0.965	< 0.001 (HS)	0.552	0.16 (NS)
Pleural ADA	–	–	0.38	< 0.001 (HS)	–	–	–	–	–	–

*TPE* Tuberculous pleural effusion, *MPE* pleural effusion

There is a significant positive correlation between serum and pleural IL-33 levels in all groups of the study and each group; tuberculous, malignant and parapneumonic pleural effusions but not in transudative pleural effusion. Pleural IL-33 positively correlates with pleural ADA in patients with tuberculous pleural effusions, (*r* = 0.38, *P* < 0.001)

The concentrations of IL-33 in the pleural effusion of the tuberculous group (22.5 ± 0.90 ng/l) were considerably more than that of the malignant group (14.6 ± 2.35 ng/l; P < 0.001) or that of parapneumonic group (7.33 ± 0.32 ng/l; P < 0.001) and transudative group (7.53 ± 0.38 ng/l; P < 0.001), Table 4.

**Table 4:** Comparison between pleural IL-33 levels in each studied groups

Effusion	Range	Mean	Paired “t”	P
Pleural IL-33 in TPE *N* = 36	21.0–23.95	22.5 ± 0.90	10.7	< 0.001 (HS)
Pleural IL-33 in MPE *N* = 33	10.32–17.32	14.6 ± 2.35		
Pleural IL-33 in Parapneumonic group *N* = 24	6.44–8.43	7.33 ± 0.72	39.7	< 0.001 (HS)
Pleural IL-33 in transudative group *N* = 24	7.12–8.21	7.53 ± 0.38	34.9	< 0.001 (HS)
Serum IL-33 in TPE *N* = 36	12.46–15.11	13.90 ± 0.85	6.73	> 0.05 (NS)
Serum IL-33 in MPE *N* = 33	15.21–20.32	13.50 ± 1.98		

*TPE* Tuberculous pleural effusion, *MPE* malignant pleural effusion

This table shows that pleural IL-33 is significantly higher in tuberculous pleural effusion than in malignant, parapneumonic, and transudative pleural effusion groups

The serum IL-33 level was statistically significantly higher in the tuberculous group (13.90 ± 0.85 ng/L) than that of parapneumonic (6.22 ± 0.72 ng/l; *P* < 0.001) group and transudative group (6.10 ± 0.42 ng/l; *P* < 0.001). However, there was no significant difference between serum IL-33 in tuberculous (13.90 ± 0.85 ng/L) and malignant (13.5 ± 1.98 ng/L; *P* >  0.05) groups, Table 5.

**Table 5:** Comparison between serum IL-33 levels in each studied groups

Effusion	Range	Mean	Paired “t.”	P
Serum IL-33 in TPE *N* = 36	12.46–15.11	13.90 ± 0.85	6.73	> 0.05 (NS)
Serum IL-33 in MPE *N* = 33	15.21–20.32	13.50 ± 1.98		
Serum IL-33 in Parapneumonic group *N* = 24	5.23–7.21	6.22 ± 0.76	20.5	< 0.001 (HS)
Serum IL-33 in transudative group *N* = 24	5.38–6.67	6.10 ± 0.42	23.9	< 0.001 (HS)

*TPE* Tuberculous pleural effusion, *MPE* malignant pleural effusion

This table shows that serum IL-33 is significantly higher in tuberculous pleural effusion than of parapneumonic and transudative pleural effusion groups. However, there was no significant difference between serum IL-33 in tuberculous and malignant groupse

**Fig. 1:**
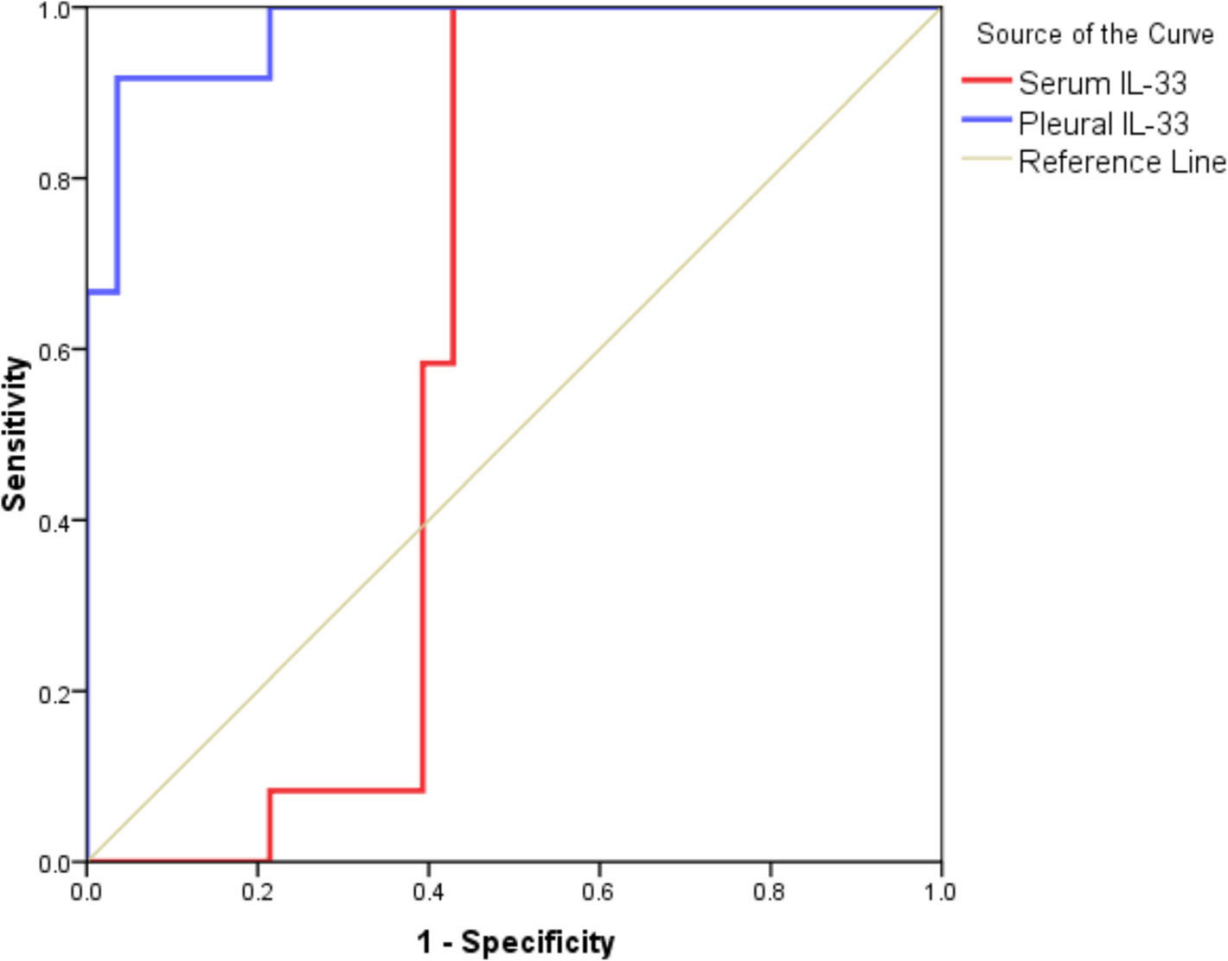
ROC curve for the performance of serum and pleural IL-33 in the prediction of tuberculous effusion

A 19.16 ng/l was the cutoff point of pleural IL-33 level for the diagnosis of tuberculous pleural, with a sensitivity of 91.7% and specificity of 96.4%. The serum IL-33 level of tuberculous pleural effusion group was between 9.83–15.16 ng/l, with a sensitivity of 100% and specificity of 96.4% Table 6, Fig. 1.

**Table 6:** Cutoff value, sensitivity, specificity, the positive predictive value of serum and pleural IL-33 level in the prediction of tuberculous pleural effusion

Variable	Cutoff	Sens%	Spec%	PPV%	NPV%	Accuracy%	AUC	95%CI	P
Serum IL-33 in TPE	> 9.83–15.16	100%	57.1%	50%	100%	70%	0.607	0.43–0.78	0.29 (NS)
Pleural IL-33 in TPE	> 19.16	91.7%	96.4%	91.7%	96.4%	95%	0.973	0.92–1.0	< 0.001 (HS)
Combined		100%	96.4%	92.3%	100%	97.5%	0.982	0.94–1	< 0.001 (HS)

*TPE* Tuberculous pleural effusion, MPE malignant pleural effusion

This table shows that the cutoff value of pleural IL-33 level for the diagnosis of tuberculous pleural effusion is 19.16 ng/l. So IL-33 levels more than this threshold give a high probability of being tuberculous pleural effusion. The area under the corresponding ROC curve (AUC) was 0.973. The 95% confidence interval is 0.92–1, the corresponding sensitivity and specificity are 91.7 and 96.4% (*P* < 0.01). Pleural and serum IL-33 level combination increase the sensitivity to 100% and specificity to 96.4%

**Fig. 2:**
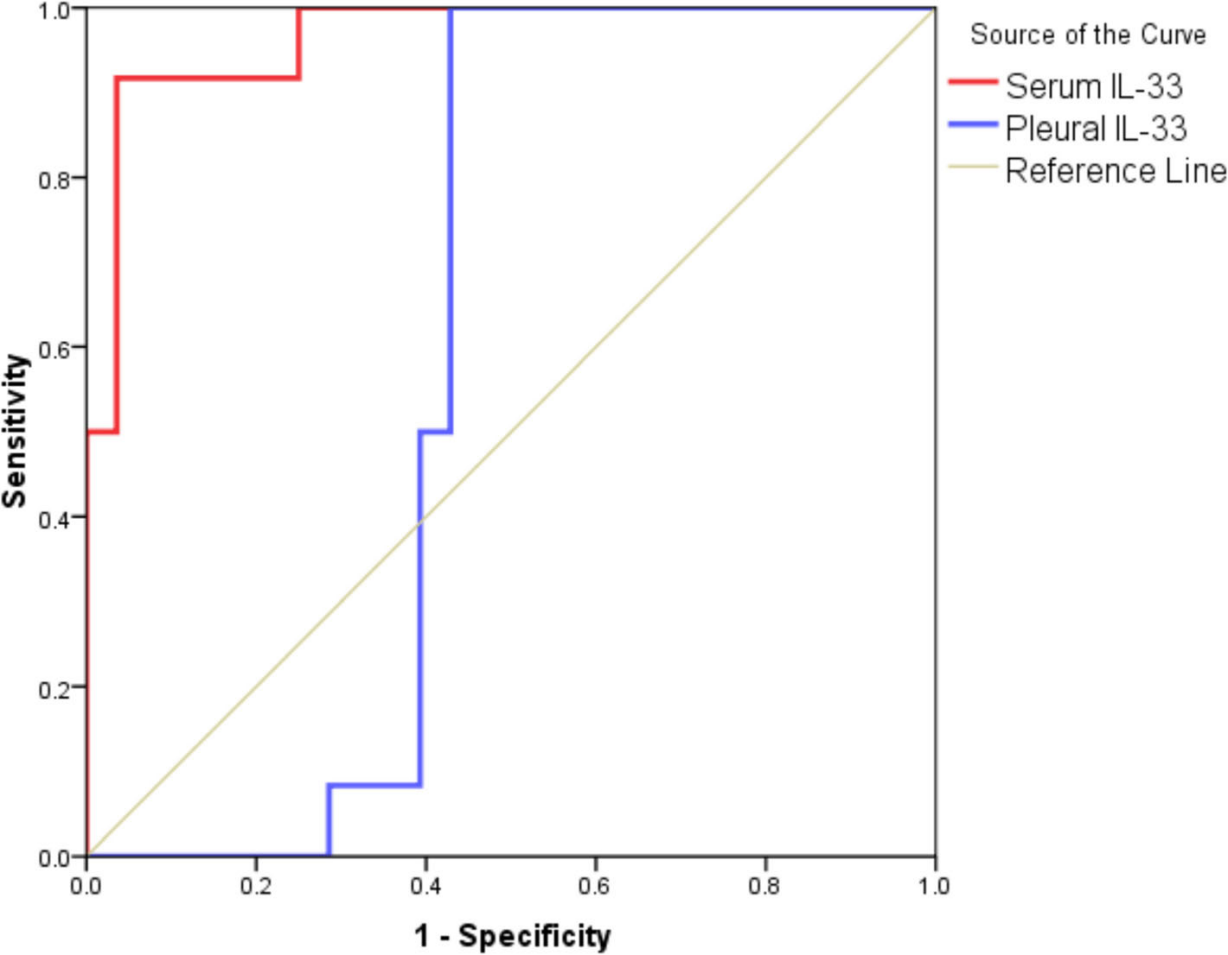
ROC curve for the performance of serum and pleural IL-33 in the prediction of malignant effusion

The cutoff point of the pleural/ serum IL-33 ratio for the diagnosis of tuberculous ‘pleural effusion was > 1.4; with a sensitivity of 91.7% and specificity of 100%. The cutoff point of the pleural/serum IL-33 ratio for the diagnosis of malignant pleural effusion was < 0.9, with a sensitivity of 83.3% and specificity of 96.4%, Table 7, Fig. 2.

**Table 7:** Cutoff value, sensitivity, specificity, positive predictive value, and negative predictive value of pleural/serum IL-33 ratio in the prediction of tuberculous effusion

Variable	Cutoff	Sens%	Spec%	PPV%	NPV%	Accuracy%	AUC	95%CI	P
Pleural/serum ratio	> 1.4	91.7%	100%	100%	96.5%	97.5%	0.94	0.85–1.0	< 0.001 (HS)
Pleural/serum ratio	< 0.9	83.3%	96.4%	90.9%	93.1%	92.5%	0.90	0.77–1.0	< 0.001 (HS)

The cutoff value of pleural/serum IL-33 ratio for the diagnosis of tuberculous pleural effusion was > 1.4. Therefore patients with pleural/serum IL-33 ratio > 1.4 had a high probability of having a tuberculous pleural effusion. The value for the area under the corresponding ROC curve (AUC) was 0.94. The 95% confidence interval was 0.85–1.0; the corresponding sensitivity and specificity were 91.7 and 100% (*P* < 0.001). The cutoff value of pleural/serum IL-33 ratio for the diagnosis of malignant pleural effusion is < 0.9, therefore patients with pleural/serum IL-33 ratio < 0.9 has a high probability of having a malignant pleural effusion. The value for the area under the corresponding ROC curve (AUC) is 0.90. The 95% confidence interval was 0.77–1.0, the corresponding sensitivity and specificity were 83.3and 96.4% (*P* < 0.001)

## Discussion

IL-33 has been suggested to have a role in the pathogenesis of pleural inflammation and effusion [19]. IL-33 may serve as a novel biomarker to differentiate pleural effusions, especially tuberculous from malignant effusions. The pleural fluid IL-33 levels were significantly higher of the corresponding serum IL-33 levels in all our studied groups moreover, it was considerably higher in each subgroup, however, serum IL-33 levels in the malignant group were significantly higher than the corresponding pleural level and might be used to differentiate it from other causes of pleural effusions Table 2. This significant serum-pleural IL-33 difference of the malignant group could be explained by that the tumor development results in down-regulation of IL-33 in epithelial cells but up-regulation of IL-33 in the tumor stroma and serum [8].

In this study, the positive correlations of pleural effusions IL-33 concentrations with the corresponding serum samples of all patients of the study groups (r = 0.677, *P* < 0.001); moreover, the positive correlation of subgroups; pleural IL33 in tuberculous, malignant and parapneumonic pleural effusion groups with their corresponding serum IL33 level (*r* = 0.848, *P* < 0.001; *r* = 0.881, *P* < 0.001; and *r* = 0.965, *P* < 0.001) respectively was in agreement with Xuan et al., Lee et al. and Li et al., who in their studies found that; the concentrations of IL-33 in the pleural effusions of tuberculous, malignant and parapneumonic groups positively correlated with that of the corresponding serum samples [17, 20, 21]. ,Also, pleural IL-33 was positively correlated with pleural ADA in patients with tuberculous pleural effusions, (*r* = 0.38, *P* < 0.001), Table 3, in accordance to koung et al. and Lee et al. who demonstrated statistically significant positive correlations between IL-33 levels and ADA [20]. This positive correlation makes the test more reliable in diagnosing tuberculous pleural effusion.

In this study, the significant higher pleural and serum IL-33 concentrations in tuberculous pleural effusion group more than other groups, Table 4, and the non-significant difference found between serum IL-33 in tuberculous and malignant (*P* >  0.05) groups, Table 5, strongly points to tuberculous etiology and could be used to differentiate it from malignant cause. Similarly, Xuan et al., Lee et al. and Li et al. demonstrated tuberculous pleural effusion IL-33 levels were significantly higher than malignant pleural effusion IL-33 levels [17, 20, 21]. Xuan et al. and Li et al. also found a non-significant difference between serum IL-33 in tuberculous and malignant groups [17, 21]. However, Lee et al., disagree these results and found that levels of serum IL-33 (26 pg/mL) significantly higher in patients with tuberculous pleural effusion than that of malignant pleural effusion (16 pg/mL, *P* = 0.03) [20].

The cutoff point of the studied pleural IL-33 levels for the diagnosis of tuberculous pleural effusion was; 19.16 ng/l with 91.7% sensitivity and 96.4% specificity. The serum IL-33 level of the tuberculous group was between 9.83–15.16 ng/l; therefore patients with serum and pleural level of IL-33 at these ranges had a high probability of being diagnosed with tuberculous pleural effusion with a sensitivity of 100% and specificity of 96.4%, Table 6. While the cutoff point of the pleural/ serum IL-33 ratio for the diagnosis of tuberculous ‘pleural effusion was > 1.4; therefore patients with pleural/serum IL-33 ratio > 1.4 had a high probability of being diagnosed with tuberculous pleural effusion with 91.7% sensitivity and 100% specificity. The cutoff point of the pleural/serum IL-33 ratio for the diagnosis of malignant pleural effusion was < 0.9; therefore patients with pleural/serum IL-33 ratio < 0.9 had a high probability of being diagnosed with malignant pleural effusion with 83.3% sensitivity and 96.4% specificity, Table 7.

Xuan et al. found that the cut-off value of pleural IL-33 for the diagnosis of tuberculous pleural effusion was; 19.86 ng/l with 86.96% sensitivity and 90.48% specificity [17]. Li et al. found that the area under curve (AUC) of IL-33 to differentiate tuberculous pleural effusion from all non- tuberculous pleural effusions 0.823 (95% CI, 0.737–0.909) with a cut-off value of 68.3 pg/ml, the sensitivity and specificity were 83.9 and 70.9%, respectively [21].

Ni Zeng and colleges carried out a systematic review over 38 studies to study the interleukins value for diagnosis of tuberculous pleural effusion, and found that the sensitivity, specificity for the selected ILs were: IL-2, 0.67and, 0.76; IL-6, 0.86 and, 0.84; IL-12, 0.78 and, 0.83; IL-12p40, 0.82 and, 0.65; IL-18, 0.87 and, 0.92; IL-27, 0.93 and, 0.95 [22]. Wang and colleges had two prospective studies and one meta-analysis study to assess the diagnostic accuracy of IL-27 in tuberculous pleural effusion; in Beijing study, they reported 96.1% sensitivity and 99.0% specificity with an area under the curve of 0.983 when the cut-off value was 591.4 ng/L. Also, the superior diagnostic accuracy of interleukin 27 reported by Wuhan cohort study and confirmed in their meta-analysis study [23]. These results were comparable to the pleural IL-33 level in the tuberculous pleural effusion of this study (91.7% sensitivity and 96.4% specificity).

This study has some limitations as; a small number of patients used to confirm the diagnostic role of IL-33, so broader scale study on a large number of patients is needed to verify the diagnostic role of IL-33. This study was carried out in a high prevalence TB area (Egypt); other studies are required to assess low TB prevalence populations, and how would their results differ from high TB prevalence populations? IL-33, like many different biomarkers, cannot be relied on for definitive diagnosis and pleural intervention is still needed to confirm the diagnosis. However, IL-33 can be used as a diagnostic aid in cases of pleural effusions to guide further investigations as it is a stable and affordable biomarker that can be measured in serum and pleural effusion, our data supports its use as a novel biomarker in a high prevalence population, and further study in low prevalence populations is warranted.

## Conclusion

the present study showed that the mean pleural IL-33 levels were significantly higher than that of serum in all study groups except in the malignant group where serum levels were higher than pleural levels. The cutoff point of pleural IL-33 level for the diagnosis of tuberculous pleural effusion was 19.16 ng/l with sensitivity of 91.7%, specificity of 96.4% and the cutoff point of pleural/ serum IL- 33 ratio for diagnosis of tuberculous pleural effusion was > 1.4 with sensitivity of 91.7% and specificity of 100% and for the determination of malignant pleural effusion was < 0.9 with sensitivity of 83.3% and specificity of 96.4%. Pleural IL-33 is a novel, fast (4.5 h), reliable biomarker assay for the differentiation of pleural effusion especially tuberculosis from the malignant pleural effusion.

## Data Availability

The datasets used and/or analysed during the current study are available from the corresponding author on reasonable request.

## Abbreviations

ADA: Adenosine deaminase
AFB: Acid-fast bacilli
AUC: Area under the curve
CBC: Complete blood count
CT: Computed tomography
ESR: Erythrocyte sedimentation rate
IFN-γ: Interferon- Gamma
IL: Interleukin
LDH: Lactate dehydrogenase
MPE: Malignant pleural effusion
NF-κB: Nuclear factor κ-light-chain-enhancer of activated B cells
ng/l: Nanogram/litre
T.B: Tuberculosis
Th1: Type 1 helper cell
Th2: Type 2 helper cell
TNF: Tumor necrosis factor
TPE: Tuberculous pleural effusion
